# Commentary on ‘accelerating clinical development of HIV vaccine strategies: methodological challenges and considerations in constructing an optimized multi-arm phase I/II trial design’

**DOI:** 10.1186/1745-6215-15-76

**Published:** 2014-03-13

**Authors:** Dennis Dixon

**Affiliations:** 1 20814-6043 Bethesda, Maryland, USA

## 

The well-written manuscript has lead me to reflect on the present state of the art of designing early trials, not necessarily limited to trials of investigational vaccines. Standard trial designs are inadequate for this trial, as the authors demonstrate by their careful literature search and review. In fact, more and more research plans seem to need one or another departure from standard designs. Maybe the traditional pdigm of choosing a design that: (a) has been ‘credentialed’ by publication in a peer-reviewed methodology journal; and (b) is as close as possible to matching the actual research objectives of the investigators, even if not a precise match, is obsolete.

Richert et al. illustrate a new pdigm, which may well be their real contribution. They summarize what is already known about the various effects of the candidate vaccine strategies. They carefully state what new knowledge they seek. They describe the proposed trial with all its specifications and assumptions, including those needed for them to study the design’s statistical properties. They describe the simulation study they performed, in enough detail that others could reproduce it, and tabulate the results. In fact, not only could others undertake to reproduce their results, it is clear how to proceed to study other specifications and assumptions.

What are the implications of following the new pdigm rather than the old one? Two come to mind readily. With regard to peer review of the clinical trial, evaluation of the design under the old pdigm would very often end with an observation that the proposed study employs a well-established plan as published by Gehan [1] or Simon [2] or Thall and Cheng [3] (and so on). Under the new pdigm, that would almost never suffice, and competent, serious review by a statistical scientist would be needed. I note that, in the U.S., at least, Institutional Review Boards (IRBs) (ethics review) have to address the validity of the science of each project, but many IRBs lack statistics expertise. The situation may be better in the context of reviewing funding applications, although peer reviewers rarely see full protocols in final form.

Another implication is a dramatic decline in articles on experimental design of trials in the statistical and trial methodology literature. Each new trial would follow the pdigm, but the particulars would be essentially unique. This is a less worrisome consequence, since professional statisticians can presumably find other ways to qualify for career advancement.
